# Biallelic *NSUN3* Variants Cause Diverse Phenotypic Spectrum Disease: From Isolated Optic Atrophy to Severe Early-Onset Mitochondrial Disorder

**DOI:** 10.1167/iovs.66.6.17

**Published:** 2025-06-04

**Authors:** Neringa Jurkute, Heiko Brennenstuhl, Monika Kustermann, Lindsey Van Haute, Christian D. Mutti, Enrico Bugiardini, Takayuki Handa, Masaru Shimura, Axel Petzold, James Acheson, Anthony G. Robson, William L. Macken, Michael G. Hanna, Robert D. S. Pitceathly, Ashirwad Merve, Urania Kotzaeridou, Stefan Kölker, Michael Freilinger, Marcus Erdler, Reginald E. Bittner, Johannes A. Mayr, Yasushi Okazaki, Kei Murayama, Holger Prokisch, Andrew R. Webster, Michal Minczuk, Gavin Arno, Berthold Pemp, Georg F. Hoffmann, Wolfgang M. Schmidt, Patrick Yu-Wai-Man

**Affiliations:** 1 Moorfields Eye Hospital NHS Foundation Trust, London, UK; 2 Institute of Ophthalmology, University College London, London, UK; 3 Department of Neuro-ophthalmology, The National Hospital for Neurology and Neurosurgery, University College London Queen Square Institute of Neurology, The National Hospital for Neurology and Neurosurgery, London, UK; 4 Department of General Pediatrics, Division of Pediatric Neurology and Metabolic Medicine, Heidelberg University Hospital, Heidelberg, Germany; 5 Institute of Human Genetics, Heidelberg University, Germany; 6 Neuromuscular Research Department, Center for Anatomy and Cell Biology, Medical University of Vienna, Vienna, Austria; 7 Medical Research Council Mitochondrial Biology Unit, University of Cambridge, Cambridge, UK; 8 Department of Neuromuscular Diseases, University College London Queen Square Institute of Neurology, London, UK; 9 NHS Highly Specialised Service for Rare Mitochondrial Disorders, Queen Square Centre for Neuromuscular Diseases, The National Hospital for Neurology and Neurosurgery, London, UK; 10 Department of Pediatrics, Toho University Ohashi Medical Center, Tokyo, Japan; 11 Institute of Neurogenomics, Helmholtz Zentrum München, Neuherberg, Germany; 12 Center for Medical Genetics, Department of Metabolism, Chiba Children's Hospital, Chiba, Japan; 13 Neuro-ophthalmology Expert Center Amsterdam, Amsterdam UMC (Locatie VUmc), Amsterdam, Netherlands; 14 Department of Neuropathology, The National Hospital for Neurology and Neurosurgery, London, UK; 15 Department of Paediatrics and Adolescent Medicine, Neuropediatrics, Medical University of Vienna, Vienna, Austria; 16 Department of Neurology, Klinik Donaustadt, Karl-Landsteiner-Institut, Vienna, Austria; 17 Department of Pediatrics, University Hospital Salzburg, Paracelsus Medical University, Salzburg, Austria; 18 Diagnostics and Therapeutic of Intractable Diseases, Intractable Disease Research Center, Graduate School of Medicine, Juntendo University, Tokyo, Japan; 19 Laboratory for Comprehensive Genomic Analysis, RIKEN Center for Integrative Medical Sciences, Kanagawa, Japan; 20 Center for Medical Genetics, Department of Metabolism, Chiba Children's Hospital, Chiba, Japan; 21 School of Medicine, Institute of Human Genetics, Technische Universität München, Munich, Germany; 22 Institute of Neurogenomics, Computational Health Center, Helmholtz Munich, Neuherberg, Germany; 23 German Center for Child and Adolescent Health (DZKJ), partner site Munich, Munich, Germany; 24 North Thames Genomic Laboratory Hub, Great Ormond Street Hospital for Children NHS Foundation Trust, London, UK; 25 Department of Ophthalmology and Optometry, Medical University of Vienna, Vienna, Austria; 26 Cambridge Eye Unit, Addenbrooke's Hospital, Cambridge University Hospitals, Cambridge, UK; 27 John van Geest Centre for Brain Repair and MRC Mitochondrial Biology Unit, Department of Clinical Neurosciences, University of Cambridge, Cambridge, UK

**Keywords:** *NSUN3*, optic atrophy, inherited optic neuropathy, tRNA methylation, mt-tRNA modification, mitochondrial disorder

## Abstract

**Purpose:**

Primary mitochondrial disorders (PMDs) are a clinically heterogeneous group of genetic disorders that can affect many tissues, with a broad phenotypic spectrum ranging from isolated organ involvement to severe early-onset multisystem disease. Visual loss from optic atrophy is a frequent clinical manifestation of mitochondrial cytopathies. This study aimed to identify the missing heritability in previously unsolved cases of suspected isolated or syndromic optic neuropathy. Based on three recent reports on biallelic *NSUN3* variants causing early-onset PMD, we explored in detail the genetic and clinical spectrum of *NSUN3*-associated disease.

**Methods:**

Affected individuals were analyzed by exome or genome sequencing. In silico variant analysis and functional assays were performed to investigate the consequences of the identified variants. Detailed phenotyping data were collected from medical records and direct questioning after the identification of candidate-likely pathogenic variants.

**Results:**

Interrogation of exome and genome sequencing data led to the identification of six candidate *NSUN3* variants in eight affected individuals from five unrelated families (including a previously reported case). A broad phenotypic spectrum was observed ranging from isolated optic atrophy to severe early-onset PMD. Identified *NSUN3* variants impairing NSUN3 activity are located within the S-adenosylmethionine-dependent methyltransferases domain and loss of function variants were associated with a more severe phenotype. Remarkably, bilateral optic atrophy was a unifying clinical feature observed in almost all affected individuals.

**Conclusions:**

Pathogenic or likely pathogenic biallelic variants in *NSUN3* disrupt mt-tRNA^Met^ methylation and mitochondrial translation leading to mitochondrial disease ranging from mild isolated optic atrophy to a severe multisystemic phenotype with possible limited life expectancy.

Primary mitochondrial disorders (PMDs) are caused by pathogenic variants in either the mitochondrial or nuclear genome (mtDNA, nDNA), leading to perturbed oxidative phosphorylation (OXPHOS) or other aspects of mitochondrial functioning.^1^^–^^3^ The majority of these disorders have an overlapping clinical phenotype, necessitating a broad approach to molecular diagnostic testing, typically including full sequencing of mtDNA and use of large virtual gene panels.^4^^,^^5^ Mitochondrial tRNA genes account for ∼60% of mtDNA genes; therefore mutations in tRNA and tRNA-modifying proteins are a major cause of PMD.^5^ Biallelic variants in the tRNA modifier *NSUN3* were recently reported as a cause for early-onset severe PMD characterized by combined mitochondrial respiratory chain complex deficiency.^6^^–^^8^ *NSUN3* encodes NOP2/Sun RNA methyltransferase 3, NSUN3 protein (mitochondrial tRNA (cytosine(34)-C(5))-methyltransferase) essential for mt-tRNA^Met^ methylation (5-methylcytosine [m^5^C]) at the wobble base C34.^7^ This modification is necessary for a subsequent oxidation and m^5^C conversion to 5-formylcytosine (f^5^C), required for normal mitochondrial translation and function.^6^ Deficiency of mt-tRNA^Met^ m^5^C has been shown to result in reduced f^5^C and defective mitochondrial translation, oxidative phosphorylation, and respiratory function. To date, only two affected individuals from unrelated families have been reported to carry biallelic pathogenic *NSUN3* variants with one report missing functional data.^8^^–^^10^ This study reports previously unreported candidate pathogenic variants in *NSUN3* and provides detailed phenotyping and functional data confirming the role of *NSUN3* in mitochondrial disease.

## Material and Methods

### Study Cohort

Patients and families were identified from a genetically unsolved cohort of individuals having had prior investigation for variants in genes known to cause inherited optic neuropathy or PMD. The index family (Family 1, GC 25895) was identified through the inherited eye disease clinics at Moorfields Eye Hospital NHS Foundation Trust (London, United Kingdom [UK]). An additional UK family (Family 2, GC 29835) was identified through the UK's 100,000 Genomes Project (100KGP) recruited at University College London Queen Square Institute of Neurology and The National Hospital for Neurology and Neurosurgery (London, UK). Family 3 was identified through the collaboration with the Neuro-Ophthalmology Clinic at the Department of Ophthalmology of the Medical University of Vienna (Vienna, Austria). Family 4 was identified through a collaboration with Chiba Children's Hospital (Chiba, Japan) and Toho University Ohashi Medical Center (Tokyo, Japan). Family 5 was identified through GeneMatcher (https://genematcher.org).^11^ The latter family was previously reported.^8^ This study adhered to the Declaration of Helsinki and all contributing centers had ethical and institutional approvals: Moorfields Eye Hospital NHS Foundation Trust and the Northwest London Research Ethics Committee (Genotype and Phenotype in Inherited Neurodegenerative Disease [REC 13/YH/0310], Genetic Study of Inherited Eye Disease [REC 12/LO/0141], UK Genomics England 100,000 Genomes project [REC 14/EE/1112]) and the Medical University of Vienna (EC-numbers: 1566/2022 and 1969/2021). Written informed consent was given from all participants, their parents, or legal guardians.

### Clinical Phenotyping

Medical records were reviewed following the identification of candidate *NSUN3* variants to establish the phenotypic spectrum of *NSUN3*-associated disease. All affected individuals underwent a comprehensive ophthalmological and neurological examination during the diagnostic workup or after the molecular diagnosis.

### Molecular Genetic Analysis

Genome sequencing (GS) or exome sequencing (ES) were implemented to identify candidate genotypes in affected individuals. The proband from Family 1 (GC 25895) had GS performed as part of the 100KGP as previously described.^12^^,^^13^ A multistep rare variant filtering pipeline was used: First, rare variant filtering (minor allele frequency [MAF] <0.001) for protein altering variants in genes previously shown to be associated with inherited optic neuropathy (https://panelapp.genomicsengland.co.uk/panels/186/) was performed, which did not identify any candidate variant/s.^12^ Because the family was consanguineous, an autosomal recessive disease was suspected, and the search was broadened to include potential biallelic homozygous protein-altering variants across the entire genome with MAF <0.001, which revealed a rare homozygous variant in *NSUN3*. To identify additional individuals carrying biallelic *NSUN3* variants, GS dataset analysis was performed across all previously unsolved cases recruited into 100KGP (pilot and main studies).

In family 3, ES was performed on the proband's blood-derived DNA sample. DNA libraries were prepared using the SureSelect Clinical Research Exome V2 (Agilent Technologies, Winooski, VT, USA) enrichment protocol and sequenced on an Illumina NovaSeq6000 sequencing system (150 bp paired-end reads, at 60-fold average read depth on target). All library preparation and sequencing steps were performed at GATC Biotech AG (Constance, Germany; now Eurofins Genomics). Sequences were analyzed at the Neuromuscular Research Department using an in-house developed bioinformatics pipeline, starting from FASTQ files. In brief, reads were mapped to the human genome reference sequence (build hg19) using the BWA-MEM algorithm. Post-alignment processing steps and variant calling were performed using the genome analysis toolkit. Variant annotation and comparison to reference databases (1000 genomes project, NHLBI GO exome Sequencing Project, gnomAD 2.1, and an in-house database comprising >1500 individuals) was conducted using ANNOVAR and exported to Excel spreadsheets for final analysis steps and variant prioritization. Filtering for potentially damaging variants in genes known to be causatively involved in optic atrophy and fulfilling a MAF cutoff <0.001 in reference datasets resulted in no candidate. Because no potentially pathogenic variant was found in a gene with known relevance for isolated optic atrophy and the vast majority (>99%) of the variants identified were homozygous on chromosome 3, the entire region/all genes on that chromosome were further screened for potential pathogenic variants.

In family 4, ES was performed using genomic DNA (gDNA) isolated from patient's blood. Indexed gDNA libraries were prepared from gDNA and exomes were captured using SureSelect V6 exome enrichment kits (Agilent Technologies), in accordance with the manufacturer's protocol. Sequencing was performed using 150-bp paired-end reads on a HiSeq4000 (Illumina). The quality of raw data was checked by FASTQC. After removing the low-quality reads and adaptors, reads were mapped to the reference genome (GRCh38/hg38) with Burrows-Wheeler Aligner, Picard, and SAMtools. The genome analysis toolkit was also used for insertion and deletion realignment, quality recalibration, and variant calling. Detected variants were annotated using both ANNOVAR and custom Ruby scripts. Variants were filtered with MAFs of >0.5% for dbSNP, 1KG and the Genome Aggregation Database (gnomAD).

Subsequently, research findings were submitted to GeneMatcher (https://genematcher.org), which led to collaboration with another site, and the identification of the affected individual from Family 5. Bidirectional Sanger sequencing using standard reagents and protocols was performed to confirm segregation in available relatives (primers available on request).

An additional mitochondrial DNA (mtDNA) sequencing was performed for affected individuals who had ES performed. For proband from the family 3 DNA was isolated from skeletal muscle tissue using the Qiagen QiAamp DNA Mini Kit protocol. Mitochondrial DNA was amplified by long-range PCR (using adjacent primers binding to positions m.6103 to m.6160 located in the MT-CO1 gene: forward primer: 5′-CTT TGG CAA CTG ACT AGT TCC CCT AAT-3′; reverse primer: 5′-CCT CCG ATT ATG ATG GGT ATT ACT ATG AAG A-3′). DNA libraries were prepared from purified long-range PCR products and sequenced on an Illumina NovaSeq6000 sequencing system (150 bp paired-end reads) at an output of ∼5 million reads, resulting in an average per-base depth of ∼50,000-fold. Reads were mapped to the revised Cambridge Reference Sequence (rCRS, GenBank NC_012920) and variant calling and annotation was performed using the Mutserv software.^14^^,^^15^

### In Silico Analyses

To predict the functional impact of missense variants, in silico Mutation Taster (http://www.mutationtaster.org) predictive algorithms were applied. Candidate variants were annotated based on the ACMG/AMP guidelines using the VarSome prediction tool (accessed February 2022).^16^ The evolutionary conservation of the affected amino acid residues across orthologs was assessed using HomoloGene (https://www.ncbi.nlm.nih.gov/homologene).

### Biochemical Studies/Functional Assays

To demonstrate mitochondrial tRNA methylation at m5C34 wobble base, total RNA was purified from PAXgene-stabilized whole blood from five affected individuals (three affected individuals from Family 1 and two affected individuals from Family 3). In addition, tRNA methylation was investigated on fibroblasts obtained from the proband from Family 2. RNA bisulfite sequencing (RNA-BisSeq) was performed as previously described.^8^ For comparison, age- and gender-matched unrelated healthy individuals (*n* = 4) samples were studied.

### Cell Culture and Immunocytochemistry

Primary human dermal fibroblasts (dFs) were grown, fixed, and stained as described previously.^17^ Confocal microscopy was performed by using an Olympus FLUOVIEW FV3000 confocal microscope equipped with PlanApoN 60 × 1.4 NA and UPLAN FLN 40 × 1.3 NA objective lenses (Olympus, Tokyo, Japan). Images were recorded using the Olympus FluoView software, and processed and analyzed using ImageJ software (NIH, Bethesda, MD, USA).

### Antibodies

The following primary antibodies (Abs) were used for immunocytochemistry (ICC,) Western blotting (WB), or both: mouse monoclonal antibodies (mAbs) to cytochrome c oxidase subunit I (Cox I; ICC: 1:100; Molecular Probes, Eugene, OR, USA), OxPhos Human WB Antibody Cocktail (WB: 1:1000; Invitrogen, Carlsbad, CA, USA), and β-Actin (WB: 1:1000; Sigma-Aldrich Corp., St. Louis, MO, USA); rabbit mAb to Tom20 (ICC: 1:200, WB: 1:1000; Cell Signaling Technology, Danvers, MA, USA), and chicken polyclonal Ab to Vimentin (WB: 1:10000; Novus Biologicals, Littleton, CO, USA). For ICC, primary antibodies were used in combination with donkey anti-rabbit IgG Alexa Flour 555, donkey anti-mouse IgG Alexa Flour 647 (all from Invitrogen) and Phalloidin-Atto 488 (ICC: 1:350, Sigma-Aldrich). Nuclei were counterstained with 4,6-Diamidin-2′-phenylindol-dihydrochlorid (DAPI; Sigma-Aldrich). For immunoblot analyses HRP-conjugated secondary antibodies (Dako, Glostrup, Denmark) were used in combination with ECL Select Western blotting detection reagent (Amersham, Piscataway, NJ, USA).

### Preparation of Cell and Tissue Lysates, SDS-PAGE, and Western Blot Analysis

Cells grown on a 15 cm dish were directly scraped off in 500 µL 6× SDS sample buffer (500 mM Tris-HCl pH 6.8, 600 mM DDT, 10% SDS, 0.1% bromophenol-blue, 30% glycerol), DNA sheared by pressing the samples through a 27 gauge needle, and samples were incubated for 10 min at 95°C. Serial 10 µm-cryosections of frozen skeletal muscle tissue (musculus vastus lateralis or musculus gastrocnemius) were homogenized in lysis buffer (pH 7.5 containing 2 mM EGTA, 2 mM EDTA, 20 mM Tris, 150 mM NaCl, 2 M urea, 3.5% SDS, and 1.5% β-mercaptoethanol), mixed with 6 × SDS sample buffer, and incubated for 10 minutes at 95°C. SDS-PAGE was performed as described.^18^ Proteins were transferred to nitrocellulose membranes (Protran 0.45 NC; Amersham) using a Mini Protean Tetra Cell blot apparatus (Bio-Rad Laboratories, Ann Arbor, MI, USA).

### Muscle Histology, Histochemistry, Transmission Electron Microscopy, and Respiratory Chain Analyses

Cryosections (10 µm) of frozen skeletal muscle tissue were stained with hematoxylin and eosin (H&E). Standard cytochrome *c* oxidase (COX) and succinate dehydrogenase (SDH) histochemical analyses were performed on skeletal muscle sections (20 µm), according to established protocols.^19^ Briefly, 20 µm sections were incubated at 37°C in a humidified chamber with either COX or SDH staining solution for 30 or 90 minutes, respectively. Excess solution was removed from the slides by washing with aqua destillata. Slides were dehydrated for two minutes in the following concentrations of ethanol: 70%, 80%, 90%, 96%, and 99.5%. Afterward, slides were additionally incubated for two minutes in 99.5% ethanol and subsequently two minutes in n-butyl acetate and mounted with eukitt (Sigma Aldrich Corp.). Activities of respiratory chain complexes (NADH:cytochrome *c* oxidoreductase, NADH:O_2_ oxidoreductase, succinate:cytochrome *c* oxidoreductase, cytochrome *c* oxidase) and citrate synthase (CS) was measured in the homogenate of the frozen muscle according to standardized protocols.^20^^–^^22^ Skeletal muscle transmission electron microscopic pictures were taken with a Jeol 1200 electron microscope (Jeol, Tokyo, Japan).

### Mitochondrial Respiratory Chain Assay of Skin Fibroblasts

Oxygen consumption rate (OCR) was measured using a Seahorse XFe96 Pro Analyzer and the Seahorse XF Cell Mito Stress Test according to the manufacturer's instructions (Agilent Technologies). The Seahorse XFe96 well plate was collagen-coated (0.1% Bovine Collagen Solution Type I (Sigma-Aldrich Corp.) 1:20 in PBS for three hours at 37°C), washed twice with PBS and dFs were seeded at a density of 1*10^4^ cells per well in 80 µL proliferation media. One hour before the assay the plate was rinsed once with non-supplemented Seahorse XF DMEM medium, pH 7 (Agilent Technologies) and incubated with assay medium (Seahorse XF DMEM medium (Agilent Technologies) supplemented with 25 mM glucose (Sigma-Aldrich Corp.), 2 mM L-glutamine (Sigma-AldrichCorp.), and 1 mM pyruvate (Sigma-Aldrich Corp.) and incubated for one hour at 37°C in the absence of CO_2_. The vials of the Mito Stress Test were reconstituted with Seahorse XF DMEM assay medium before use. OCR was determined before injection of specific metabolic inhibitors and after adding 1.5 µM oligomycin (Port A), 3 µM Carbonyl cyanide 4-(trifluoromethoxy)phenylhydrazone (FCCP; Port B), and 0.5 µM rotenone/antimycin A (with Hoechst 33342 [Sanofi-Aventis, Paris, France]; Port C). After Seahorse measurement, cells were imaged and normalized to fluorescent cell counting with Hoechst using the Cytation 5 Cell Imaging Multimode Reader (BioTek, Winooski, VT, USA). Wave Pro 10.1.0 software was used to analyse Seahorse measurements.

### Quantification and Statistical Analysis

Data has been presented as the mean with a standard deviation (±SD). Statistical analysis was performed using GraphPad statistical software (version 8). Data was analyzed by one-way ANOVA followed by a Sidak test as a post hoc test for multiple comparisons.

## Results

### Molecular Genetic Analysis

**Figure 1.**
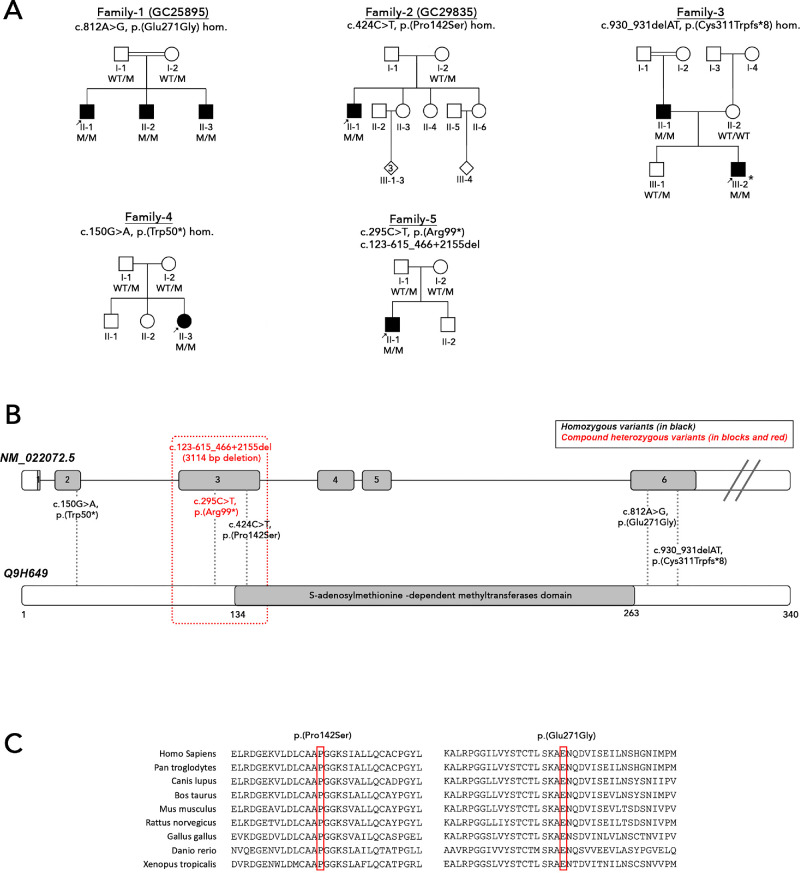
Pedigrees of families and identified candidate variants in *NSUN3*. **(A)** Pedigrees of study families. An *arrow* indicates the proband. *Shaded symbols* represent affected individuals. *Asterisk* indicates proband, III-2 from Family 3, who has a paternal uniparental isodisomy of chromosome 3. **(B)** Schematic diagram of *NSUN3* gene. Variants are indicated at the corresponding exons of the *NSUN3* gene. Homozygous variants are indicated in *black*. Compound heterozygous variants are indicated in *red* and *blocks*. *Double asterisk* indicates previously reported genotypes. (**C**) Multiple alignment of NSUN3 orthologs. Affected residues are strictly conserved in mammalian orthologs. M, mutant; WT, wildtype.

Independent genetic studies led to the identification of candidate genotypes in eight affected individuals from five unrelated families (Fig. 1A), with one individual previously reported (II-1, Family 5).^8^ Six distinct candidate variants were identified in *NSUN3* (NM_022072, Table 1; Fig. 1A, B), of which two, c.150G>A, p.(Trp50*) and c.295C>T p.(Arg99*), were considered to be loss-of-function (LOF). Two variants, c.424C>T p.(Pro142Ser) and c.812A>G p.(Glu271Gly), were missense. Two variants, c.123-615_466+2155del p.(Glu42Valfs*11) and c.930_931delAT p.(Cys311Trpfs*8), were frameshift variants affecting two different exons. Analysis of variant distribution showed that variants can be found across the whole *NSUN3* gene/protein, with only one homozygous missense variant c.424C>T p.(Pro142Ser) being located directly within the S-adenosylmethionine-dependent methyltransferases domain (SAM-domain region 134 – 263 amino acid residues), affecting the S-adenosylmethionine binding site (Fig. 1B). All variants were rare or absent from the gnomAD dataset (Table 1). A paternal uniparental isodisomy of chromosome 3 was identified in III-2 from Family 3 leading to homoallelism for c.930_931delAT.

**Table 1.** Genotypes Identified in the Study Cohort

Family	No. of Affected	HGVSc	HGVSp	GT	gnomAD v2.1.1. (MAF)	Exon	In Silico	ACMG/AMP (Accessed February 2022)
Family 1 GC25895	3	c.812A>G	p.(Glu271Gly)	1/1	0.000007958	6	Disease causing	VUS (PM2, PP1, PP3)
Family 2 GC29835	1	c.424C>T	p.(Pro142Ser)	1/1	0.00003193	3	Disease causing	LP (PM2, PP3, PS3)
Family 3	2	c.930_931delAT	p.(Cys311Trpfs^*^8)	1/1^*^	—	6	Disease causing	VUS (PM2, PP1)
Family 4	1	c.150G>A	p.(Trp50^*^)	1/1	0.00001994	3	Disease causing	P (PVS1, PM2, PP3)
Family 5	1	c.123-615_466+2155del	p.(Glu42Valfs^*^11)	0/1	*—* *^†^*	3	Disease causing	P (PVS1, PS3, PM2, PPP3, PM3, PP5)
		c.295C>T	p.(Arg99^*^)	0/1	*0.00003182*	3	Disease causing	LP (PM2, PM3, PM4, PP5)

ACMG/AMP, American College of Medical Genetics and Genomics/Association for Molecular Pathology; GT, genotype; HGVS, Human Genome Variation Society and HGVS notations (see at http://varnomen.hgvs.org); HGVSc, the HGVS coding sequence name; HGVSp, the HGVS protein sequence name; LP, likely pathogenic; P, pathogenic; VUS, variant of uncertain significance.

All variants reported by using NM_022072.5 as reference according to HGVS nomenclature.

* Both father and son are homozygous for the variant. The father is homozygous because of consanguinity, whereas the son became homozygous because of paternal uniparental isodisomy.

† A rare structural variant, 3:93802331-93805448del, with breakpoints close to the identified variant? is found on 1 allele (MAF 0.00004610) in gnomAD dataset.

Because all members of Family 1, except the father, were noticed to have large optic discs and cupping, cavitary optic disc anomaly (CODA) was considered. To exclude CODA, the GS data was reviewed for the presence of the previously reported^23^ intergenic triplication or any other type of CNV at the associated locus (12q), which was negative. In addition, proband II-1 and his youngest brother II-3 were identified to have blue dot cataract (Supplementary Fig. S1). This was not observed in II-2. To identify additional ocular genetic condition, a cataract panel was applied which did not reveal any variants in known genes.

### The Clinical Spectrum of NSUN3-Associated Disease

Affected individuals were diagnosed with isolated inherited optic neuropathy presenting as slowly progressive visual loss (Family 1 and II-1 from Family 3) or subacute Leber hereditary optic neuropathy (LHON)-like phenotype (III-2 from Family 3), or with optic atrophy associated with more severe neurological phenotypes, consistent with a PMD (affected individuals from Families 2, 4 and 5). The age of disease onset ranged from neonatal to 39 years of age. Individuals carrying biallelic LOF variants or homozygous missense variant affecting SAM binding site presented with severe neurological phenotype in early infancy. Individuals diagnosed with isolated optic atrophy presented in adolescence with the youngest individual diagnosed at the age of 11 years. Bilateral optic neuropathy/atrophy was noted in the majority (7/8, 87.5%) of affected individuals. A male (*n* =7) predominance was observed in our study cohort.

**Figure 2.**
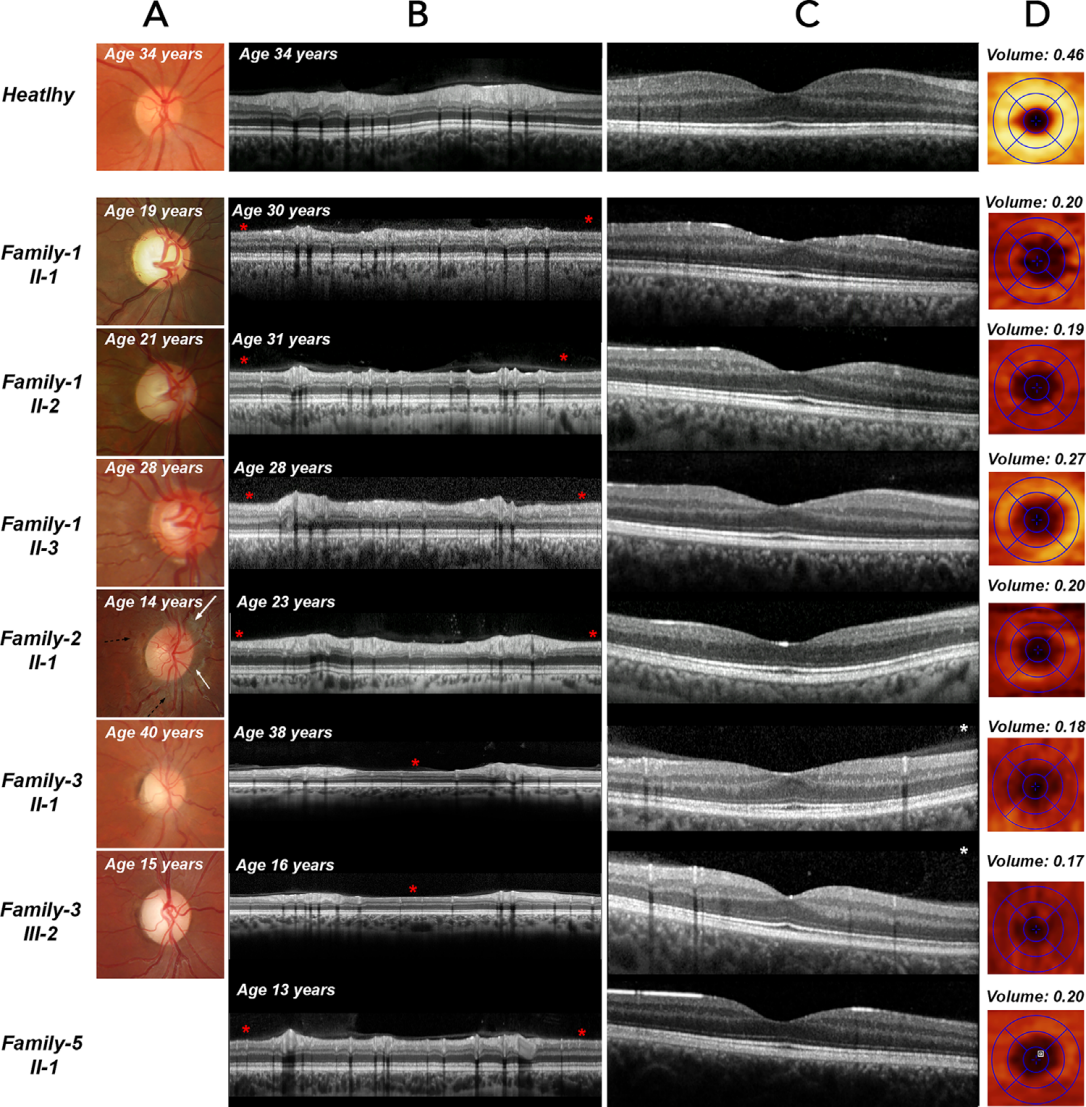
Multimodal imaging composite of affected individuals (right eye only). **(A)** Optic nerve head color photographs demonstrate various degrees of optic nerve pallor in all affected individuals (color fundus photograph is not available for the affected individual from Family 5). **(B)** OCT B-scan of the peripapillary area showing RNFL thinning (*red asterisks*) within the temporal segment of the optic nerve head in all affected individuals. **(C)** OCT B-scan through the macula area demonstrating RNFL and GCL thinning in all affected individuals. **(D)** RGC layer thickness maps overlaid with macula grid (1.0, 2.22, 3.45 mm diameters) on SD-OCT scans. RGC thickness heatmaps indicate thinning of the RGC layer in all affected individuals. Multimodal imaging composite of a healthy individual is provided for comparison on the top of the image composite.

All affected individuals from Family 1 (GC 25895) are born to consanguineous parents of Pakistani origin. All of them were found to have bilateral optic atrophy, diagnosed at the age of 15–16 years (proband, II-1), 18–19 years (II-2), and 25–26 years (II-3). Fundus examination revealed large optic nerve heads and cupping with subtle temporal optic disc pallor (Fig. 2A) that was confirmed by Spectralis spectral-domain optical coherence tomography (SD-OCT) in all affected family members. SD-OCT of the optic nerve showed marked peripapillary retinal nerve fiber layer (pRNFL) thinning within the temporal quadrant (Fig. 2B). Macular scans indicated pronounced RNFL, retinal ganglion cell (RGC) layer thinning bilaterally (Fig. 2C) and reduced RGC volume (Fig. 2D). Despite mild hearing impairment in II-1 and II-3 (currently under investigation), no other systemic or neurological symptoms were present at the time of investigation. Neuroimaging studies of all affected individuals of Family 1 showed bilateral optic neuropathy with no intracranial abnormalities.

Proband from Family 2 (II-1, GC 29835) is born to non-consanguineous parents from Colombia. He was diagnosed with sideroblastic anemia around age one month. Systemic investigations revealed mild developmental delay with strong preservation of language skills, microcephaly, short stature, and cardiac abnormalities with bicuspid aortic valve and atrial septal defect. Endocrinologic evaluation showed growth hormone deficiency, insulin resistance, and borderline thyroid function. Neurological assessment indicated generalized axonal sensorimotor neuropathy and mild sensorineural hearing loss. At the age of 14 years, he developed a bilateral optic neuropathy with fundus changes typically seen in LHON. Fundus imaging (Fig. 2A) showed hyperemic edematous appearance of the optic discs, swelling of the pRNFL (Fig. 2A, white arrows), and characteristic telangiectatic microangiopathy (Fig. 2A, black arrows) with increased tortuosity of the central retinal vessels. At the age of 24 years he underwent SD-OCT imaging, which revealed pRNFL thinning (Fig. 2B), RNFL, and RGC layers thinning bilaterally (Figs. 2C, 2D). He was also found to have left amblyopia, exotropia, and a right-sided ptosis. Over 13 years, his visual acuity progressed from 6/6 in the right eye and 6/9 in the left eye to 6/24 and 6/36, respectively. At the age of 23 years, his brain magnetic resonance imaging (MRI) showed relative brachycephaly and hyperostosis of the skull vault (HP:0004437) with no intracranial abnormalities (data not shown). He exhibited craniofacial asymmetry (HP:0004484) considered likely to be the result of his anemia (marrow expansion). He continues to have transfusion-dependent sideroblastic anemia with severe iron overload. It is possible that the patient's anemia is not associated with candidate-likely pathogenic *NSUN3* variant. A homozygous LOF variant in a gene important for heme synthesis was identified in this patient (confidential data).

Individual 3 (Family 3: III-2), born to non-consanguineous parents of Afghani origin, presented with subacute painless bilateral vision loss at the age of 11 years. During the initial presentation visual acuity was 6/60 in the right eye and 6/120 in the left eye. Optic discs of both eyes showed mild temporal pallor, inferior and superior pRNFL swelling, and tortuosity of peripapillary vessels. His MRI study was unremarkable. Over the following three months, his visual acuity decreased to 6/120 in both eyes, with increasing central scotomas and loss of pRNFL in OCT. Although OCT showed further loss of pRNFL and ganglion cell layer (GCL) until 32 months from onset (Fig. 2B), visual acuity began to improve spontaneously after 12 months and reached 6/12 after a further 18 months. Subsequently, oral treatment with idebenone was started, whereupon visual acuity was restored to 6/6 in both eyes after only four months. Visual field testing also showed improvement at that time. Neurological and hearing assessments were normal. Because the clinical picture was suggestive of LHON, genetic testing for the three most common LHON mtDNA variants followed by whole mtDNA sequencing was performed. Primary genetic testing analysis did not identify pathogenic variants that could account for the disease. Subsequently ES was performed, and data analysis revealed a candidate variant of uncertain significance in homozygous state: *NSUN3* c.930_931delAT p.(Cys311Trpfs*8). Segregation studies identified the same genotype in the father, who was found to have moderate bilateral optic atrophy (Fig. 2B) with reduced visual acuity in the left eye. All other family members (mother and brother) showed no signs of optic nerve disease. Further analysis revealed paternal uniparental isodisomy of chromosome 3 in the proband's DNA sample. Both affected family members had blue-dot cataracts, normal lactate, but elevated homocysteine levels.

Individual 4 (Family 4: II-3) was born at 38 weeks to non-consanguineous parents of Japanese origin. Until the age of one year, she met her motor milestones, followed by delayed walking until the age of two years. She showed inadequate weight gain and last weighed 12 kilograms at four years of age, when she exhibited a first epileptic seizure. Medical examination revealed hypoglycemia (glucose measured at 39 mg/dL), hypernatremia (sodium levels measured at 118 mEq/L), and metabolic acidosis (pH 7.144, pCO_2_ 16.3 mmHg, HCO3^-^ 5.6 mmol/L, Lac 1.3 mmol/L). Neuroimaging showed T2 hyperintense lesions and swelling in the left parietal and occipital lobes (data not shown). CSF studies were unremarkable. Stool sample analysis found her to be positive for norovirus. Despite levetiracetam treatment, she continued to experience seizures about once every six months. At the age of nine years, she experienced a convulsive seizure followed by transient ocular symptoms (Visual impairment and abnormal ocular movements. No information on optic nerves appearance is available.), dysarthria, and muscle weakness. ES studies identified homozygous c.150G>A p.(Trp50*) *NSUN3* variant.

Individual 5 was previously reported in 2016 by Van Haute and colleagues.^8^ He presented at the age of three months with failure to thrive, global developmental delay with muscular hypotonia, and low-grade microcephaly. Convergence nystagmus was noticed during the course of the disease. Brain MRI imaging (including spectroscopy) and electroencephalography scans of the patient were unremarkable. OCT imaging revealed thinning of pRNFL and GCL layers, which showed progressive changes over the course of the disease (longitudinal data not shown). Because of moderately elevated plasma lactate, further investigation of an underlying mitochondriopathy was performed with a muscle biopsy, which revealed a combined OXPHOS deficiency. Pathogenic and likely pathogenic variants in *NSUN3* were reported, c.123-615_466+2155del and c.295C>T p.(Arg99*). Further functional investigation revealed the impact of NSUN3 on epitranscriptome modification, mitochondrial protein synthesis and respiratory function.^8^ The clinical information for individuals carrying candidate *NSUN3* genotypes have been summarized in Table 2.

**Table 2.** Clinical Manifestations of Study Subjects

Family	Individual, Sex	Age of Onset and Initial Presentation	Ophthalmological Features (Age)	Extraocular Features CNS/PNS	Other	MRI (Age at Study)	Associated HPO Terms
Family 1 GC25895	II-1, M	14–15 years;	Optic atrophy	Mild hearing impairment		Slender optic nerves (21 years)	HP:0007663
		Reduced visual acuity	Blue dot cataract				HP:0000648
			Visual acuity (30 years): RE 6/12 and LE 6/18				HP:0012712
			Color vision, Ishihara (30 years): RE 16/17 and LE 12/17				HP:000064
	II-2, M	18 years;	Optic atrophy		SAM levels in serum: 81 nmol/L	Slender optic nerves and slightly T2 hyperintense (21 years)	HP:0000648
		Reduced visual acuity	Visual acuity (30 years): BE 6/12		(range 86 – 145 nmol/L)		HP:0000642
			Color vision, Ishihara (30 years): BE 14/15				
	II-3, M	25–26 years;	Optic atrophy	Mild hearing impairment	SAM levels in serum: 83 nmol/L	Slender optic nerves (27 years)	HP:0000648
		Reduced visual acuity	Blue dot cataract		(range 86 – 145 nmol/L)		HP:0012712
			Visual acuity (26 years): BE 6/19				
			Color vision, Ishihara (26 years): BE 17/17				
Family 2 GC29835	II-1, M	One month;	Optic atrophy (LHON-like presentation)	Mild hearing impairment	Short stature	Relative brachycephaly and hyperostosis of the skull vault (23 years)	HP:0001903
		anemia	Left exotropia	Generalized axonal sensorimotor neuropathy	Sideroblastic anemia		HP:0001112
			Amblyopia	Microcephaly	Cardiac abnormalities: bicuspid aortic valve, ASD		HP:0001924
			Right-sided ptosis	Developmental delay	Endocrinopathies: growth hormone deficiency, insulin resistance and borderline thyroid function		HP:0011342
			Visual acuity (24 years): RE 6/6 and LE 6/9	Peripheral neuropathy	Bilateral ankle deformities.		HP:0000252
							HP:0004322
							HP:0001647
							HP:0001631
							HP:0000824
							HP:0000855
							HP:0007141
							HP:0012712
							HP:0001138
							HP:0000646
							HP:0000577
							HP:0007687
							HP:0000248
Family 3	II-1, M	40 years;	Optic atrophy		Elevated plasma homocysteine	Cerebral small vessel disease (Grade 1) (40 years)	HP:0000648
		Reduced visual acuity	Blue dot cataract				
			Visual acuity (40 years): RE 6/6 and LE 6/12				
			Impaired color vision (desaturated panel D-15 test)*				
	II-2, M	11 years;	Optic atrophy (LHON-like presentation)		Elevated plasma homocysteine	Normal (12 years)	HP:0000648
		Reduced visual acuity	Blue dot cataract				HP:0001112
			Visual acuity (15 years): BE 6/6.				
			Impaired color vision (desaturated panel D-15 test)*				
Family 4	II-3, F	4 years:	Seizure-associated visual impairment (9 years) and abnormal eye movements (9 years)	Failure to thrive	Metabolic acidosis	T2 hyperintensities and edema in the left parietal and occipital lobes (4 years)	HP:0001270
		Seizure, reduced consciousness		Developmental delay	Hypoglycemia		HP:0001508
				Seizure-associated dysarthria			HP:0001250
				Seizure-associated muscular hypotonia			HP:0000505
							HP:0000496
							HP:0001260
							HP:0001252
Family 5	II-1, M	<3 months:	Optic atrophy	Failure to thrive	Small for gestational age	Normal (12 years)	HP:0001508
		Developmental delay, growth restriction	Convergence nystagmus. Visual acuity (15 years): RE 6/9 and LE 6/19 Impaired color vision*	Fine motor impairment Developmental delay	Increased serum lactate Lactic acidosis		HP:0001263 HP:0001252 HP:0000252 HP:0000642 HP:0000639 HP:0000639

ASD, atrial septal defect; BE, both eyes; CNS, central nervous system; F, female; LE, left eye; M, male; PNS, peripheral nervous system; RE, right eye.

* Impaired color vision was documented but no further details on number of identified plates is available.

### Visual Electrophysiology Findings

#### Family 1

**Figure 3.**
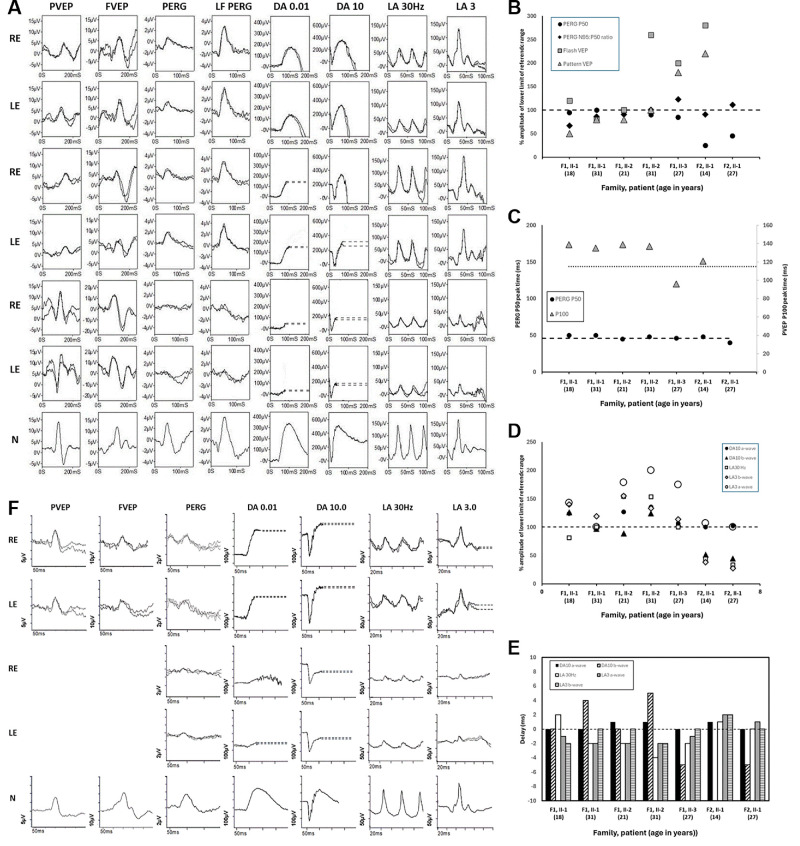
Electrophysiological recordings. **(A)** International-standard pattern and flash VEP (PVEP; FVEP), pattern ERG, large field pattern ERG (LF PERG) and full-field ERG recordings from the right and left eyes (RE; LE) of the proband from family 1 (II-1; rows 1 and 2), his sibling (II-2; rows 3 and 4), and from the proband from family 2 (II-1; rows 5 and 6) with representative reference recordings from one eye of an unaffected individual for comparison (row 7). Pattern ERGs were recorded to a standard stimulus field (12° × 15°) and to a large stimulus field (24° × 30°). Full-field ERGs were recorded to Xenon-based flashes and were recorded with gold foil corneal electrodes. **(B)** Pattern ERG and VEP amplitudes in four subjects including both baseline and follow-up data for II-1 and II-2 (family 1) and II-1 (family 2). The amplitudes of the PERG P50, the PERG N95:P50 ratio, pattern VEP and flash VEP amplitudes are plotted as a percentage of the lower limit of the (“normal”) reference range (*broken horizontal line*). Data showed a high degree of interocular symmetry and are shown for right eyes only. The age of the patient at the time of testing is indicated on x-axis (within *parentheses*). **(C)** Pattern ERG P50 peak times (primary y axis) and pattern VEP P10 peak times (secondary axis) in four subjects including baseline and follow-up data for II-1 and II-2 (family 1) and II-1 (family 2). The lower limit of peak time for the reference range is shown for the pattern ERG P50 component and the upper limit of peak time is shown for the pattern VEP P100 component (horizontal broken line). Data showed a high degree of interocular symmetry and are shown for right eyes only. The age of the patient at the time of testing is indicated on x-axis (within *parentheses*). **(D)** Full-field ERG amplitudes in four subjects including both baseline and follow-up data for II-1 and II-2 (family 1), and II-1 (family 2). Baseline and follow-up full-field ERGs in II-1 and II-2 (family 1) and baseline recordings in II-1 (family 2) were recorded to Xenon-based flashes, and recordings from II-3 (family 1) and follow-up recordings in II-1 (family 2), were recorded to LED-based flashes, all with gold foil corneal electrodes. To allow direct comparison of all recordings to different sources of light, amplitudes of the main ISCEV Standard ERG components are plotted as a percentage of the age-matched lower limit of the (“normal”) reference range for each component of each stimulus system (*horizontal line* indicates the lower limit of the amplitude range). Data showed a high degree of interocular symmetry and are shown for right eyes only. The age of the patient at the time of testing is indicated on x-axis (within *parentheses*). **(E)** Full-field ERG peak times in four subjects including both baseline and follow up data for II-1 and II-2 (family 1), and II-1 (family 2). Baseline and follow-up full-field ERGs in II-1 and II-2 (family 1) and baseline recordings in II-1 (family 2) were recorded to Xenon-based flashes, and recordings from II-3 (family 1) and follow-up recordings in II-1 (family 2) were recorded to LED-based flashes, all with gold foil corneal electrodes. To allow direct comparison of all recordings to different sources of light, peak times of the main ISCEV Standard ERG components are plotted as delay, with the upper limit of normality indicated by the *horizontal broken line*. Data showed a high degree of interocular symmetry and are shown for right eyes only. The age of the patient at the time of testing is indicated on the x-axis (within *parentheses*). **(F)** International-standard pattern and flash VEP (PVEP; FVEP), pattern ERG, and full-field ERG recordings from the right and left eyes (RE; LE) of II-3 (family 1) and follow-up ERGs from II-1 (family 2) with representative reference recordings from one eye of an unaffected individual for comparison (row 5). Pattern ERGs were recorded to a standard stimulus field (12° × 15°). Full-field ERGs were recorded to LED-based flashes and were recorded with gold foil corneal electrodes. Patient traces are superimposed to demonstrate reproducibility. *Broken lines* replace blink artefacts in ERGs for clarity.

Visual electrophysiology testing at the age of 18 years and 31 years (II-1), 21 and 31 years (II-2), and 27 years (II-3) was performed according to the International Society for Clinical Electrophysiology of Vision (ISCEV) standards.^24^^–^^26^ Representative electrophysiological recordings in II-1 and II-2 are shown in Figure 3A and are quantified in Figures 3B–E. Pattern VEPs were delayed and subnormal in both. Pattern ERG P50 showed borderline abnormal shortening of P50 peak time, the descending limb of the P50 component had a shallow slope, most pronounced in II-1 (Fig. 3A), and the N95:P50 ratio was subnormal in both at baseline. These findings are consistent with bilateral optic nerve/retinal ganglion cell dysfunction in both siblings. There was no evidence of worsening optic nerve function at follow-up in either case. Full-field ERGs revealed no consistent abnormality at baseline, but dark-adapted strong flash ERG (DA10) b-waves showed delay at follow-up in both eyes of both cases (by 4–5 ms; Fig. 3E), consistent with very mild inner retinal rod system dysfunction. In II-3, pattern and flash VEPs were within the reference range (Figs. 3B, 3C, 3F). The retinal recordings were relatively noisy because of the patient having some difficulties tolerating the stimuli, but pattern ERG P50 showed borderline abnormal shortening of peak time (Fig. 3C) similar to cases II-1 and II-2, and suggestive of retinal ganglion cell dysfunction. Full-field ERGs revealed no clinically significant abnormality (Figs. 3D, 3E, 3F).

#### Family 2

At the age of 14 years, electrophysiological testing revealed PERG and LF PERG P50 reductions, in keeping with macular dysfunction (Figs. 3A, 3B); Pattern ERG 50 was of borderline abnormal short peak time. Full-field ERGs were markedly abnormal, revealing an electronegative strong flash (DA) ERG, delayed and subnormal LA30Hz ERG and single flash cone (LA3) ERG with a markedly subnormal b/a ratio. The ERG findings were indicated generalized rod and cone system involvement, with a locus of dysfunction that was post-phototransduction or inner retinal bilaterally (Figs. 3A, 3D, 3E, 3F). The pattern VEPs were delayed and likely influenced by optic nerve and/or macular dysfunction bilaterally. Repeat PERG at the age of 27 years showed slight improvement in P50 amplitude but with mild worsening (shortening) of P50 peak time, with the latter suggesting mild worsening of retinal ganglion cell function (Fig. 3B); full-field ERGs showed a high degree of stability (Figs. 3D, 3E, 3F).

#### Family 3

Proband from Family 3 had severely reduced VEP amplitudes, which remained as such after the last examination following the treatment with Idebenone.

#### Family 5

Visual electrophysiology revealed delay of P100 latency bilaterally.

### Biochemical Studies

**Figure 4.**
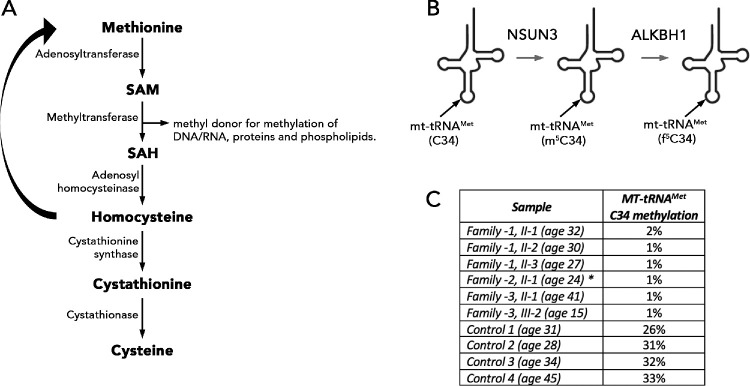
Methylation studies in study individuals. **(A)** Simplified schematic representation of methionine cycle. **(B)** Schematic representation of the formylation pathway of the wobble cytosine in mt-tRNA^Met^. NSUN3 methylates C34 and forms m^5^C34, which is further oxidized by ALKBH1 to form f^5^C34. **(C)** Methylation level at C34 wobble base was analyzed by RNA-BisSeq on blood-derived RNA, except for individual II-1 from Family 2 (indicated in *asterisk*) in whom RNA was derived from fibroblasts. Reference range 26 – 33%. ALKBH1, AlkB Homolog 1; SAH, S-adenosylhomocysteine.

All samples obtained from affected individuals demonstrated significantly reduced mitochondrial tRNA^Met^ methylation levels that were reduced to background levels, although control samples showed 26% to 33% tRNA^Met^ methylation (Fig. 4). This clearly indicated a complete loss of function of the NSUN3 protein in all these affected individuals.

### OXPHOS Activity in Skeletal Muscle and Dermal Fibroblasts

**Figure 5.**
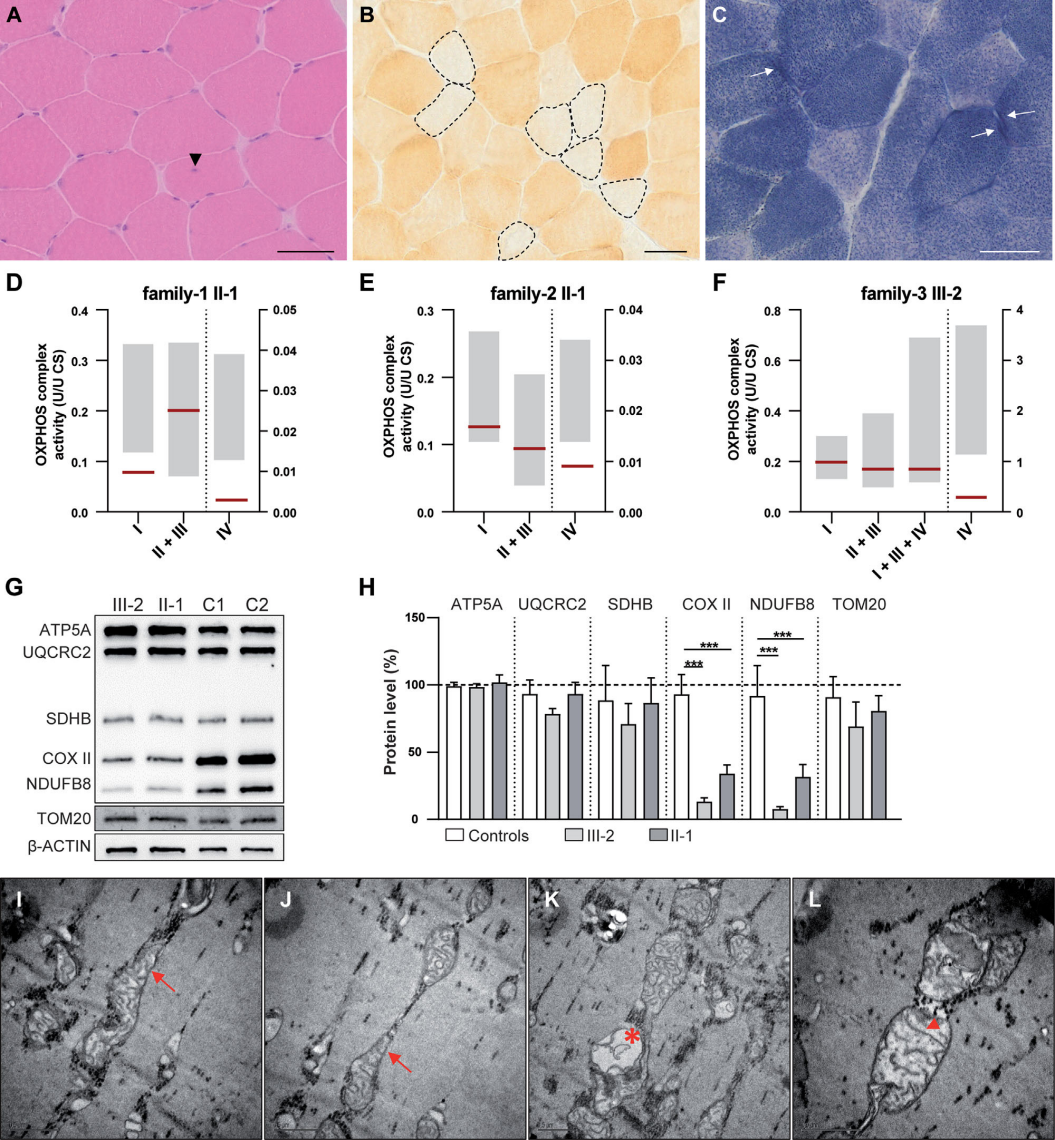
Affected individuals from Family 1, Family 2 and Family 3 displayed COX-negative fibers, diminished OXPHOS activity, and reduced amounts of some OXPHOS components in skeletal muscle. Skeletal muscle biopsy from affected individual III-2 of family 3 **(A–C)**. **(A)** H&E staining showing mild myopathic changes with rounding of muscle fibers, variation in fiber size and internal nuclei (arrowhead). **(B)** Cytochrome c oxidase (COX) staining displays fibers with markedly reduced COX activity (*dashed lines*). **(C)** Succinate dehydrogenase (SDH) staining present fibers with subsarcolemmal positive deposits (*arrows*). **(D–F)** Oxidative phosphorylation (OXPHOS) complex activity in affected individuals II-1 of family 1 **(D)**, II-1 of family 2 **(E)**, and III-2 of family 3 **(F)**. Patient muscle homogenate (*red line*) showing reduced complex IV activity for all three subjects and diminished complex I activity for patient II-2 of family 1. *Gray* *bars* represent the normal range. **(G)** Representative example of WB analysis of muscle homogenates for ATP5A, UQCRC2, SDHB, COX II, NDUFB8, TOM20 and β-ACTIN of healthy controls (C1, C2), and affected individuals III-2 and II-1 from family 3. **(H)** Quantification of 5 WB experiments of probands III-2 and II-1 from family 3 for ATP5A, UQCRC2, SDHB, COX II, NDUFB8 and TOM20 normalized to β-ACTIN. Data represent the mean value ± SD and were statistically analyzed by one-way ANOVA followed by a Sidak test as a post hoc test for multiple comparisons. **(I–L)** Representative electron micrographs depicting mitochondrial positioning in skeletal muscle of affected individual III-2. Many mitochondria were swollen and elongated, stretching the length of a sarcomere (*arrows*, **I–J**). In some fibers mitochondria were devoid of cristae (*asterisk*, **K**) and had linearized cristae membranes (*arrowhead*, **L**). *Scale bars*: 50 µm **(A–C)**, 0.5 µm **(I–L)**.

The muscle biopsy (musculus vastus lateralis) of individual 1 (Family 1: II-1) obtained at the age of 31 years showed mild nonspecific changes, including mild variation in fiber size, but no increase in internal nuclei were present (data not shown). No COX-negative fibers and no ragged red fibers were observed; however, a proportion of the type I fibers showed prominent subsarcolemmal mitochondrial aggregates (data not shown). Interestingly, spectrophotometric respiratory chain enzyme analysis demonstrated a complex I deficiency of 0.078 (normal range 0.118–0.332) and complex IV deficiency of 0.003 (normal range 0.013–0.039) (activity results are expressed normalized to citrate synthase activity; Fig. 5D). Further analysis demonstrated low muscle ubiquinone of 94 pmol/mg (normal range 140–850 pmol/mg). Ubiquinone supplementation has been commenced. Moreover, respiratory chain enzyme analysis demonstrated complex I and IV deficiencies, albeit with enzyme activities of complex II and III being within normal range (normalized to citrate synthase activity, Fig. 5D).

The muscle biopsy of the affected individual 2 (Family 2: II-1), obtained when he was 13 years of age, presented a wide variation in fiber size with atrophy and increase in endomysial connective tissue and central nuclei. Although there were no ragged red fibers, COX-negative fibers, and no excess of glycogen and lipid, some increase in tissue histiocytes was observed (data not shown). Furthermore, both fiber types (type 1 and 2) showed atrophy and grouping consistent with reinnervation (data not shown). Spectrophotometric respiratory chain enzyme analysis of the sample from the affected individual 2 demonstrated a complex IV deficiency of 0.009 (normal range 0.014–0.034, Fig. 5E).

**Figure 6.**
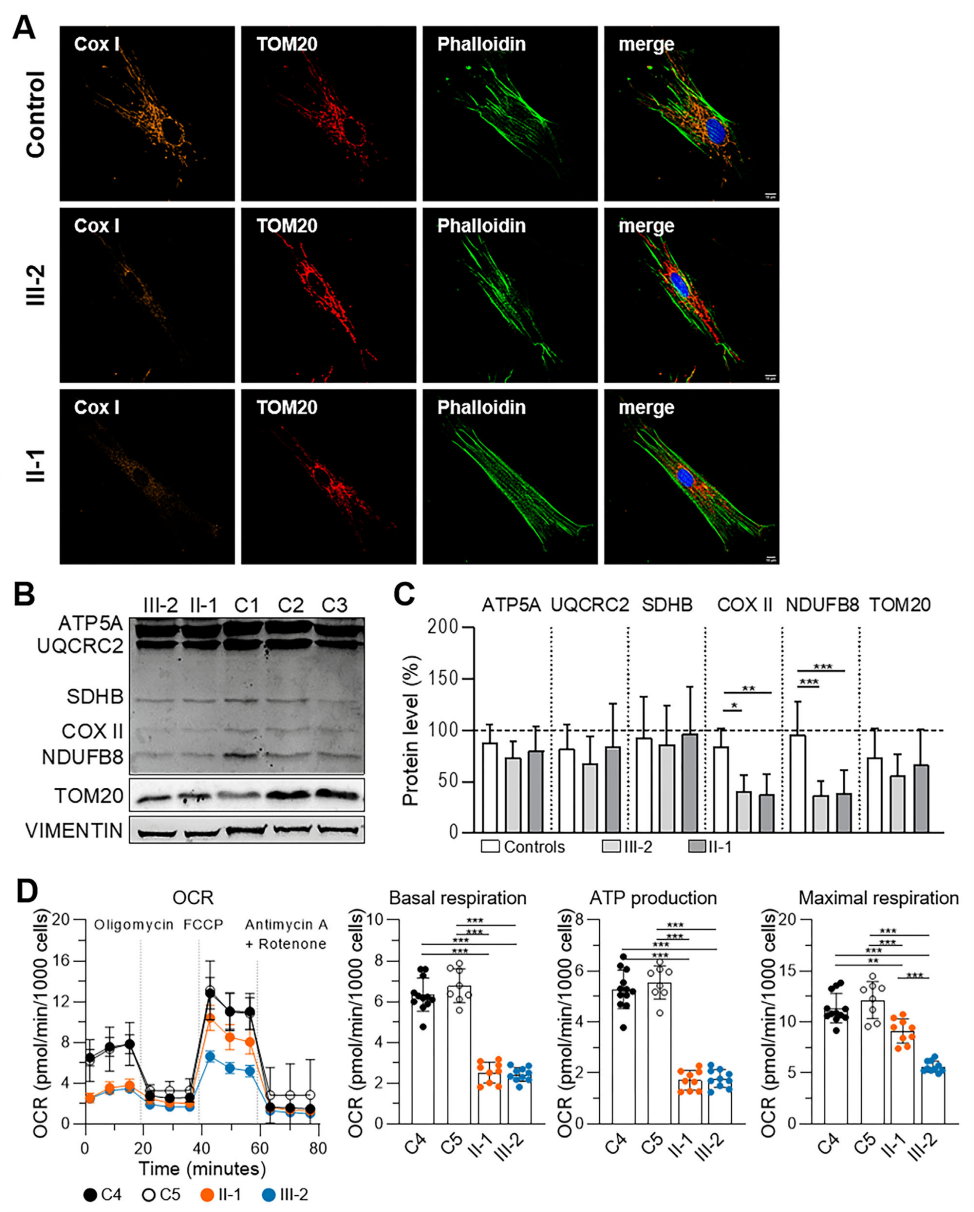
Strongly reduced COX I signals, reduced OXPHOS complex IV and I protein amounts and reduced OXPHOS activity in dermal fibroblasts of affected individuals of family 3. **(A)** Immunocytochemistry staining (ICC) of human dermal fibroblasts of a healthy control and affected individuals III-2 and II-1 of family 3. Anti-COX I (Alexa Flour 647), anti-TOM20 (AF 555), Phalloidin-Atto 488 and nuclei (DAPI). Patient fibroblasts exhibit markedly reduced COX I staining intensities. *Scale bars*: 10 µm. **(B)** Representative example of WB analysis of ATP5A, UQCRC2, SDHB, COX II, NDUFB8, TOM20 and VIMENTIN of healthy controls (C1–C3), affected individuals III-2 and II-1 of dermal fibroblast protein lysates. **(C)** Quantification of 6 WB experiments for ATP5A, UQCRC2, SDHB, COX II, NDUFB8 and TOM20 normalized to VIMENTIN. **(D)** OCR of dermal fibroblasts of healthy controls (C4, C5) and *NSUN3* patients measured with the Seahorse Cell Mito Stress Assay. OCR was recorded during sequential injections of drug inhibitors: oligomycin; FCCP; antimycin A and rotenone. Quantification of the OCR at basal respiration levels, ATP production levels and maximal respiration; cells of affected individuals II-1 and III-2 show a significant reduction of respiratory capacity and ATP production. Data represent the mean value ± SD and were statistically analyzed by one-way ANOVA followed by a Sidak test as a post hoc test for multiple comparisons.

H&E staining of skeletal muscle biopsy (musculus vastus lateralis) of individual 3 (Family 3: III-2) at the age of 14 years revealed mild myopathic changes with rounding of muscle fibers, variation in fiber size and internal nuclei (Fig. 5A). COX enzyme histochemistry demonstrated numerous COX-negative fibers (Fig. 5B). SDH enzyme histochemistry showed some subsarcolemmal mitochondrial aggregates (Fig. 5C). On the ultrastructural level, the sarcomeres, including the thick and thin filaments showed no obvious alterations; however, many mitochondria were swollen and elongated (Figs. 5I–L). In healthy muscle, mitochondria are located within the I-band, on either side of the Z-line.^27^ Instead, the individual's mitochondria were elongated, spanning the length of a sarcomere (Figs. 5I–K). Some mitochondria demonstrated massively reduced cristae numbers and linearized cristae membranes (Figs. 5K–L). Furthermore, OXPHOS deficiency was found in individual 3, especially reduced complex IV activity (0.29; normal range 1.15 - 3.69) normalized to citrate synthase activity (Fig. 5F). In line with this impaired OXPHOS activity, WB experiments of muscle lysates from affected individuals of Family 3 III-2 and II-1 (40 years) confirmed decreased amounts of COX II (OXPHOS complexes IV) and NDUFB8 (complex I), but not of complex II, III and V in the individual's skeletal muscle compared to healthy controls (Figs. 5G, 5H). As already shown in the muscle lysates, primary dFs established from III-2 and II-1 (Family 3) revealed reduced amounts of the OXPHOS proteins COX II and NDUFB8 (Figs. 6B, 6C). In addition, these dFs exhibited strongly reduced Cox I signals compared to control fibroblasts (Fig. 6A). In the next step, we examined mitochondrial function in patient-derived dFs via Seahorse XF96 respirometric analysis of the OCR and in the resting state, the OCR of dFs of affected individuals (II-1: 2.52 ± 0.52 [*n* = 9]; and III-2: 2.43 ± 0.34 [*n* = 10]) were significantly lower than in dFs of healthy controls (C4: 6.34 ± 0.81 [*n* = 12] and C5: 6.78 ± 0.83 [*n* = 8]) (Fig. 6D). Furthermore, the patient-derived cells also exhibited decreased ATP production rates (II-1: 1.71 ± 0.39 [*n* = 9] and II-2: 1.77 ± 0.35 [*n* = 10]) compared to control cells (C4: 5.28 ± 0.76 [*n* = 12] and C5: 5.55 ± 0.65 [*n* = 8]). Interestingly, after adding FCCP, a mitochondrial uncoupler that stimulates the respiratory chain to operate at maximum capacity, the OCR of the patient-derived cells (II-1: 9.11 ± 1.19 [*n* = 9] and III-2: 5.61 ± 0.54 [*n* = 10]) consistently remained below the corresponding values of control cells (C4: 11.34 ± 1.44 [*n* = 12] and C5: 12.17 ± 1.80 [*n* = 8]). Moreover, measurements of the mtDNA copy numbers in blood, skeletal muscle and dFs were unchanged compared to controls (data not shown). A skin biopsy obtained from individual 4 (Family 4: II-3) showed normal respiratory chain complex activity with reduced oxygen consumption rate (54/50% (glucose/ galactose medium) of control at maximum respiration rate) (data not shown).

## Discussion

This study has characterized the largest cohort of patients harboring candidate biallelic *NSUN3* variants to date, consisting of eight affected individuals from five independent families revealing a broad and variable phenotype. A combination of in silico variant analysis and functional assays was used to establish novel genotype-phenotype providing crucial information to inform patient management and future genetic studies for inherited optic neuropathies and PMD.

NSUN3 is required for methylation of the first wobble base at position C34 (m5C34) of mt-tRNA^Met^. Methylated cytosine undergoes further oxidation by the alpha-ketoglutarate and Fe(II)-dependent dioxygenase ALKBH1/ABH1, which results in formation of 5-formylcytosine at C34 (f5C34). In vitro studies have shown that this modification is necessary to enable optimal codon recognition during mitochondrial translation and defects in NSUN3 ultimately lead to impaired mitochondrial translation.^6^^,^^7^^,^^28^^,^^29^ Variants in *NSUN3* have been suggested as a cause mitochondrial disease. In one report, LOF variants have been found in an individual who presented at the age of three months with failure to thrive, global developmental delay and muscular hypotonia. In a second report, compound heterozygous missense variants have been identified in an early-onset encephalomyopathy with missing functional investigations.^8^^,^^9^ In both reports, variants were clustering in exon 3 and SAM domain possibly leading to a more severe phenotype.

Here, we report eight individuals from five independent families presenting with a variable phenotype of mitochondrial disease and carrying candidate biallelic variants in *NSUN3.* In addition, we provide biochemical evidence of loss of C34 mt-tRNA methylation activity and impaired complex IV activity. The age of onset of disease was highly variable, ranging from neonatal to 39 years of age (mean age at molecular diagnosis was 17 years, SD ± 12 years). Affected individuals with pronounced neurological involvement were diagnosed early in life (range 1–3 months of age at presentation), whereas individuals diagnosed with a milder phenotype, optic atrophy with or without preceding LHON-like clinical features, were diagnosed later in life (range 11–26 years of age at presentation). Optic nerve involvement was a unifying feature in most cases (7/8, 87.5%). Given that *NSUN3*-associated disease falls under the PMD group, phenotypic diversity with ocular involvement and variable disease onset are not unexpected findings.^30^^–^^33^ Particularly RGCs with their long axons and unmyelinated prelaminar segments are highly dependent on the mitochondrial-derived energy, which makes them particularly vulnerable to mitochondrial dysfunction, leading to compromised cell survival, RGC apoptosis and subsequent development of optic neuropathy/atrophy with visual loss. In several cases, good visual acuity was observed despite the presence of optic atrophy. Two individuals (II-1, Family 2 and II-2, Family 3) exhibited an LHON-like phenotype. Despite the presence of established optic atrophy, both individuals maintained very good visual acuity at the time of the most recent examination. It is well established that in classical LHON, there is a higher rate of spontaneous visual acuity improvement in subgroups experiencing childhood-onset acute vision loss compared with those with adult-onset acute LHON. However, because of the small number of cases, it is challenging to determine whether in this study this particular disease course is associated with a specific genotype or whether it is influenced by treatment with idebenone in individual II-2, Family 3. Visual improvement after subacute LHON and relatively preserved visual acuity in patients with *OPA1*-associate optic atrophy are often noted, even in the presence of significant temporal optic atrophy. This apparent discrepancy may arise if a subset of retinal ganglion cells that supply the foveola retain their functionality, thus supporting central vision.

Interestingly, although a milder clinical phenotype was observed in only five individuals, the variants in this phenotypic subgroup were located at the C-terminal end of *NSUN3*. Two of these variants (c.812A>G, p.[Glu271Gly] located downstream of the methyltransferase domain and c.930_931delAT, p.[Cys311Trpfs*8] located in the terminal exon) were predicted to evade nonsense-mediated decay, potentially leading to a truncated protein product. This may suggest that in contrast to true LOF variants or those which affect the methyltransferase domain, such variants may have a less severe effect on the affected individual. However, it should be noted that the methylation at C34 was equally disrupted in carriers of these variants, suggesting that there might be a secondary function of NSUN3 that has not yet been identified.

Electrophysiological evaluation of four affected individuals revealed clear evidence of optic nerve/retinal ganglion cell dysfunction in two siblings (II-1 and II-2, family 1) and borderline pattern ERG evidence of retinal ganglion cell dysfunction in a third (II-3, family 1). The pattern VEP abnormalities were characterized by delays and reductions, with flash VEPs showing less sensitivity. Pattern ERGs indicated a reduced N95:P50 ratio in all but one case, but all showed borderline or mild shortening of p50 peak time, an established feature of retinal ganglion cell dysfunction.^34^ The lack of VEP abnormality in II-3 (family 1) is notable, and it is tempting to speculate that pre-morbid VEPs in this individual may have been large and toward the upper limit of the amplitude reference range (i.e., the recordings may be “normal” compared with the control population but abnormal for the patient). The fourth case (II-1, family 2) had full-field ERG evidence of marked generalized retinal dysfunction, with a locus post-phototransduction or inner retinal, in addition to signs of optic neuropathy. Pattern ERG P50 was reduced, consistent with macular dysfunction, and it is highlighted that macular cone system dysfunction has been described in some cases of Leber hereditary optic neuropathy,^35^ although rarely investigated. Case II-1 (family 2) also had a complex medical history including sideroblastic anemia, life-long blood transfusions, and treatment for iron overload including use of desferrioxamine, with potentially reversible retinotoxic effects, and if it is not excluded, this may have influenced the retinal recordings. Follow-up electrophysiological data were available in three cases, and there was relative stability of function in all three, with slight enlargement of pattern ERG P50 in II-1 (family 2) after 13 years, possibly related to the complex medical history, intersession variability, or both.

Functional assays demonstrated significantly reduced mitochondrial tRNA methylation in samples obtained from six affected individuals validated pathogenicity of the identified variants. Plasma methylation profile showed a mildly reduced SAM concentration in two out of three affected individuals from Family 1 (Table 2). However, these test findings should be interpreted carefully, because measurements can be affected by diet and intake or supplementation with methionine, folate, vitamin B_12_, B_6_, betaine, and magnesium, and they are unlikely be explained solely by a single gene defect. Although there is a growing body of evidence that reduced methylation leads to cell dysfunction, with reduced SAM levels linked with cardiovascular disorders, liver diseases, cancer, developmental defects, and neurological conditions, the pathogenesis of *NSUN3*-disease could arise because of other mechanisms, and further studies are needed.^36^^–^^39^ Furthermore, the clinical potential of SAM supplementation in halting disease progression could be considered as previously reported.^40^^,^^41^

To date, genes known to harbor variants in autosomal optic neuropathy/atrophy are associated with mitochondrial organization and function (especially fission/fusion and OXPHOS), or encode complex I subunits of the mitochondrial respiratory chain.^42^^–^^46^ The two most common mitochondrial optic neuropathies are autosomal dominant optic atrophy associated with pathogenic variants in *OPA1* and LHON with three mtDNA variants (m.3460G>A, m.11778G>A and m.14484T>C) accounting for ∼90% of cases. Muscle biopsies from individuals diagnosed with *OPA1*-associated isolated or syndromic optic atrophy revealed mitochondrial myopathic features, including multiple deletions in the mtDNA, COX-deficient muscle fibers (approximately 5% and 1.3%, respectively) and numerous ragged red fibers (subsarcolemmal accumulation of abnormal mitochondria), even in the absence of a clinical myopathy.^47^^–^^49^ Some LHON patients also exhibit large subsarcolemmal mitochondrial accumulations in skeletal muscle biopsies, even when muscle weakness or atrophy is not presented.^50^^–^^53^ Furthermore, decreased complex I respiratory chain function and normal or even increased oxidative respiratory activity have also been reported.^51^^–^^53^ As seen in *OPA1*-associated disease and LHON, a heterogeneous muscle phenotype was also observed in the affected individuals carrying biallelic pathogenic *NSUN3* variants in this study. Although the majority of affected individuals (except II-1 from Family 5) did not exhibit clinical signs of myopathy, muscle biopsies demonstrated mild, nonspecific myopathic changes. Although no ragged red fibers were observed, some subsarcolemmal mitochondrial aggregates were present in three affected individuals: II-1 (Family 1), II-1 and III-2 (Family 3). Interestingly, patient II-1 (Family 1) did not exhibit COX-negative fibers, which were observed in both affected individuals from Family 3. Pathogenic *NSUN3* variants therefore cause a mitochondrial optic neuropathy with a very heterogeneous muscle phenotype, which is intriguing, given that *NSUN3* is not involved in mitochondrial organization and function, nor does it encode a complex I subunit of the mitochondrial respiratory chain.

Overall, we report novel pathogenic variants, and the highly diverse phenotypic spectrum associated with *NSUN3*-associated disease. These findings strongly suggest that pathogenic/likely-pathogenic *NSUN3* variants lead to optic atrophy with possible skeletal muscle involvement, ranging from nonspecific subclinical myopathy to a classic mitochondrial myopathy with COX-negative fibers, reduced OXPHOS activity, and mitochondrial aggregates. Establishing a confirmed molecular diagnosis in isolated and syndromic optic neuropathies can be challenging because of the wide range of causative variants in both the nuclear and mitochondrial genomes and the need for invasive investigations in some individuals, such as a muscle or skin biopsy for biochemical testing and other confirmatory functional studies. Nevertheless, nonbiased GS or ES data interrogation is a powerful tool that can be used for the discovery of novel gene-disease associations. If ES or GS is not available and even if it may be a rare cause, the *NSUN3* gene should be added to diagnostic optic atrophy and mitochondrial disease gene panels when investigating patients who are suspected to have an isolated or syndromic inherited optic neuropathy.

## Supplementary Material

Supplement 1
