# Supplementary figures and images for: Biallelic NSUN3 Variants Cause Diverse Phenotypic Spectrum Disease: From Isolated Optic Atrophy to Severe Early-Onset Mitochondrial Disorder
Source: Invest Ophthalmol Vis Sci. 2025 Jun 4;66(6):17. doi: 10.1167/iovs.66.6.17 (PMC12147050; doi:10.1167/iovs.66.6.17)

II-1

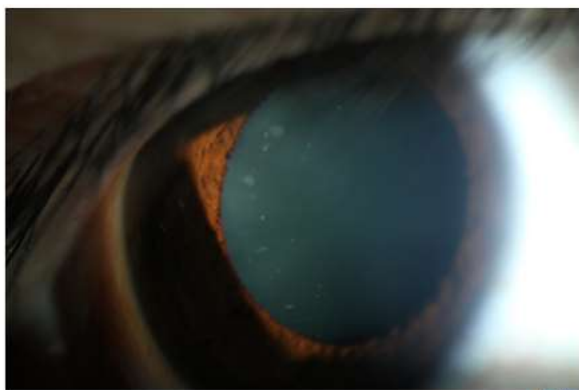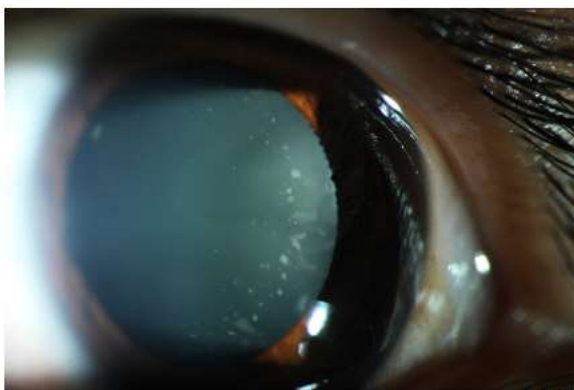

II-3

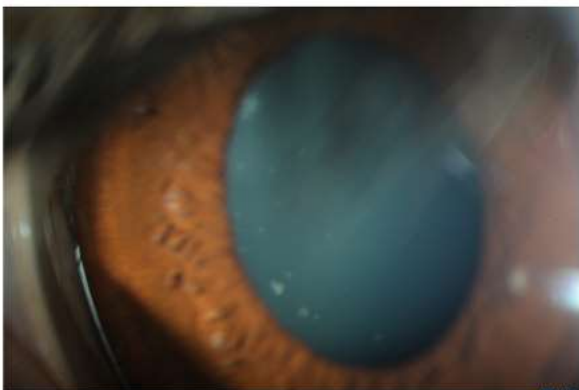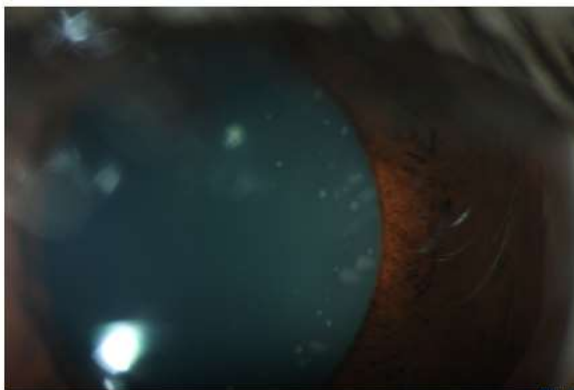

Supplement: Supplement 1 [file iovs-66-6-17_s001.pdf]
